# Influence of different great trochanteric entry points on the outcome of intertrochanteric fractures: a retrospective cohort study

**DOI:** 10.1186/s12891-017-1472-x

**Published:** 2017-03-14

**Authors:** Shuo Pan, Xiao-Hui Liu, Tao Feng, Hui-Jun Kang, Zhi-Guang Tian, Chun-Guang Lou

**Affiliations:** 1 Department of Orthopaedic Surgery, Shijiazhuang No. 1 Hospital, NO.36 Fanxi Road, Shijiazhuang, 050011 Hebei China; 2 Judicial Authentication Center of The People’s Procuratorate of Hebei Province, Shijiazhuang, 050011 Hebei China

**Keywords:** Intertrochanteric hip fracture, PFNA, Trochanteric entry point

## Abstract

**Background:**

The Proximal Femoral Nail Antirotation (PFNA) system for treatment of intertrochanteric fractures is currently widely applied worldwide. However, even though the PFNA has produced good clinical outcomes, a poor introduction technique with an inappropriate entry point can cause surgical complications. Some researchers suggest improving clinical outcomes by modifying the entry point, but no research has focused on this issue. The purpose of the present study is to compare the clinical and radiological outcomes of two different trochanteric entry points for the treatment of intertrochanteric fractures using the PFNA system.

**Methods:**

From May 2010 to October 2015, a total of 212 elderly patients with intertrochanteric fractures who were treated with the PFNA-II system were included into this retrospective cohort study. Group LA (98 patients) was treated using a lateral anterior trochanteric entry point, and group MP (114 patients) was treated using a medial posterior trochanteric entry point. All patients underwent follow-up assessments at 1, 3, 6, and 12 months after surgery. Radiographic evaluation was based on the impingement, tip-apex distance (TAD) and the position of the helical blade within the femoral head. Clinical evaluation was based on the surgical time, fluoroscopy time, blood loss, hospital stay, visual analogue scale (VAS), thigh pain, and Harris hip score.

**Results:**

The impingement was significantly reduced (*P* = 0.011) in group MP. The helical blade positions were significantly lower (*P* = 0.001) in group MP. The TADs in group LA (22.40 ± 4.43) and group MP (23.39 ± 3.60) were not significantly different (*P* = 0.075). The fluoroscopy time of group LA (53.26 ± 14.44) was shorter than that of group MP (63.29 ± 11.12, *P* = 0.000). Five iatrogenic lateral proximal fractures and 3 helical blade cutouts occurred in group LA, but none occurred in group MP. At 1 and 3 months postoperation, the Harris hip scores were significantly higher in group MP (*P* = 0.001 and *P* = 0.000, respectively), and the VAS scores were lower (*P* < 0.05).

**Conclusions:**

The medial posterior trochanteric entry point achieved excellent nail and helical blade position, reduced surgical complications, and enabled early hip function recovery but required longer fluoroscopy time than the lateral anterior trochanteric entry point.

## Background

Among elderly patients, intertrochanteric fractures are the most common osteoporotic fractures and are associated with high morbidity and mortality [1]. Due to its biomechanical advantages and rapid minimally invasive operation, the Proximal Femoral Nail Antirotation system (PFNA, Synthes, Oberdorf, Switzerland) has been more widely used to treat intertrochanteric fractures [1]. However, there is a huge difference in clinical outcome in previews studies. In his multicenter study, Simmermacher reported 8.48% implant-related complications and 5.17% reoperations in the early stage of using PFNA [2]; Wild et al. [3] has reported a cutout ratio of 7.5%, and Kristek et al.’s [4] is 1.35%. This difference is due to many factors, such as age, fracture type, implant design, fracture reduction, and implant position. Recently, an increasing number of studies have shown that a poor introduction technique leads to a poor outcome [5–7]. The entry point is the most important issue of the introduction technique. The entry point plays a vital role in the location of the PFNA after implantation and fracture reduction [8–13]. An optimal entry point can maintain fracture reduction and avoid implant-related complications.

Current research on entry points is extensive. McConnell et al. [14] emphasized that the lateral trochanteric point caused an average of 27% damage to the gluteus medius tendon during the reaming for intramedullary (IM) nail insertion. Anatomical studies of the sagittal portion of the greater trochanter tip have revealed that the entry point should be in the rear tip (0.5 cm) to accommodate the curvature of the proximal femoral medullary cavity [11].

Although entry point suggestions are reported in many articles, no retrospective study has focused on the outcomes associated with different entry points. Therefore, the objective of the present study was to compare the radiological and clinical features of patients treated by two different entry points for PFNA nail insertion. Secondary objectives identified possible causes for these outcomes and examined potential consequences for the quality of life achieved by patients with this specific implant.

## Methods

The Ethics Committee and Institutional Review Board of the Shijiazhuang No. 1 Hospital approved the retrospective study, and all patients provided informed consent.

From May 2010 to October 2015, 296 consecutive patients with intertrochanteric femoral fractures were treated with PFNA-II (PFNA Asian version; Synthes, Oberdorf, Switzerland) via the lateral anterior trochanteric entry point (group LA) or using a medial posterior trochanteric entry point (group MP). All the patients were blinded to the study, but the orthopedic surgeon in the surgical team was not. The inclusion criteria were an age ≥65 years, a low-energy injury, a closed anatomic fracture reduction and a minimum follow up of 1 year. The exclusion criteria were pathologic fractures, open fractures, delayed fractures, multiple injuries, open fracture reductions, no-anatomic fracture reductions, rheumatic diseases, immobility or walking difficulties prior to the fracture, serious medical complications, mental disorders and loss to follow up. Ultimately, 212patients (98in group LA and 114 in group MP) included in the study.

In our database general data were collected and reviewed, including the patients’ ages, sexes, American Society of Anesthesiologists (ASA) scores and Short Portable Mental Status Questionnaire (SPMSQ) scores. Hip function was recorded according to the Harris hip score. The fractures were classified through radiographs as stable or unstable according to the AO/ASIF classification by 2 surgeons (Table 1).

**Table 1:** Baseline data

		Group LA (*n* = 98)		Group MP (*n* = 114)		*P* value
Age(years)		76.64 ± 8.59		76.52 ± 8.90		0.990^a^
Gender						0.278^b^
Male		30(30.6%)		43(37.7%)		
Female		68(69.3%)		71(62.3%)		
SPMSQ						0.878^b^
Intact		49(50.0%)		61(53.5%)		
Mild		35(35.7%)		38(33.3%)		
Moderate		14(14.3%)		15(13.2%)		
ASA						0.606^b^
I		17(17.3%)		18(15.8%)		
II		32(32.7%)		36(31.6%)		
III		41(41.8%)		44(38.6%)		
IV		8(8.2%)		16(14.0%)		
Fracture side						0.835^b^
Right		34(34.7%)		38(33.3%)		
Left		64(65.3%)		76(66.7%)		
Fracture classification						0.411^b^
Stable	A1.1	8	63(64.3%)	8	67(58.8%)	
	A1.2	16		15		
	A1.3	18		21		
	A2.1	21		23		
Unstable	A2.2	17	35(35.7%)	20	47(41.2%)	
	A2.3	8		11		
	A3	10		16		
Harris Hip Score		91.70 ± 6.09		92.33 ± 5.39		0.585^c^

*SPMSQ* Short Portable Mental Status Questionnaire, *ASA* American Society of Anesthesiologists

^a^Student’ *T* test

^b^Chi-square test

^c^Mann-Whitney Test

### Surgical techniques

This retrospective study was conducted by a single center and a single surgical team. All surgical procedures were performed by senior orthopedic surgeons

All patients in both groups underwent closed anatomic fracture reduction and implantation of the PFNA-II nail, which were performed on a traction table under an image intensifier.

**Fig. 1:**
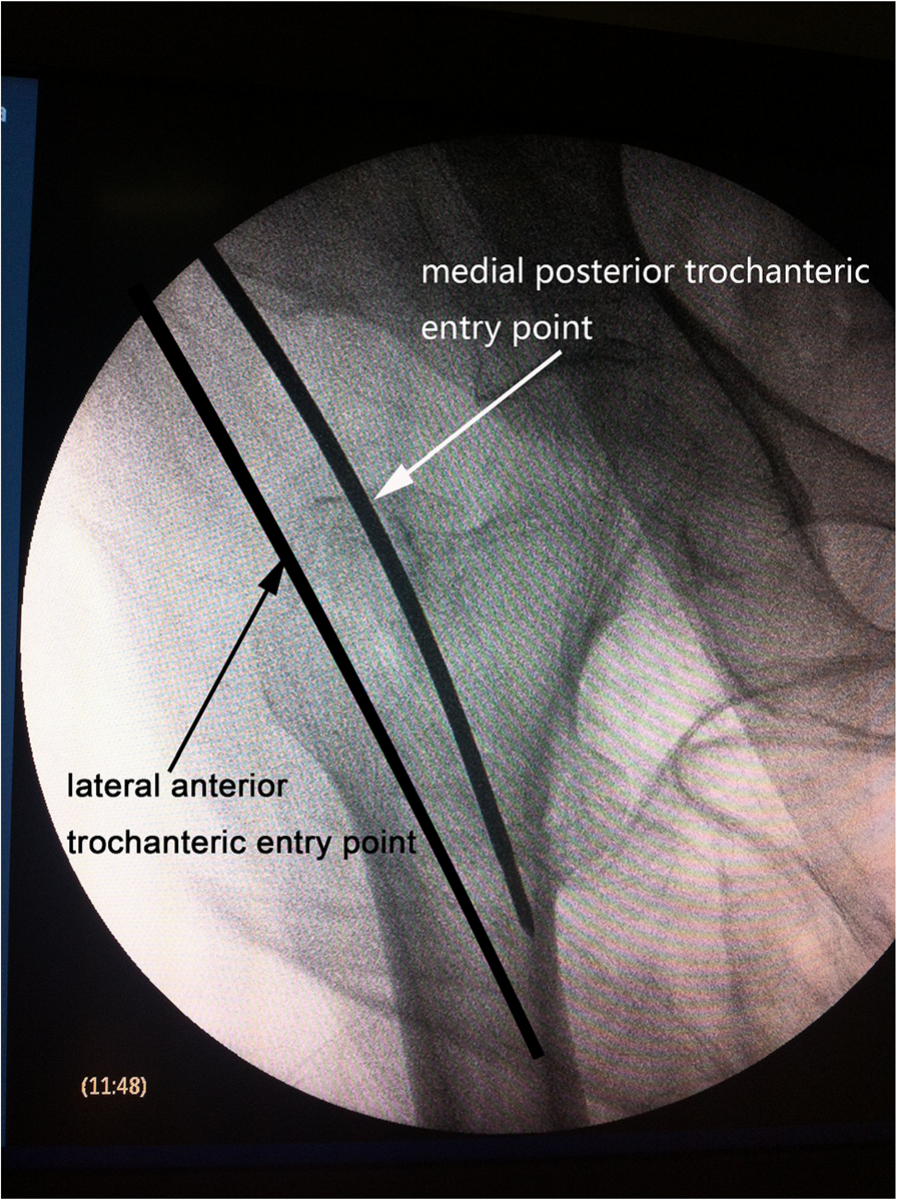
Anteroposterior radiograph. The guided pin insertion entry point in group LA was 0.5 cm lateral to the apex of the greater trochanter in the coronal plane. The guided pin insertion entry point in group MP was located 0.5 mm medial to the apex of the greater trochanter on the medial edge of the greater trochanter

**Fig. 2:**
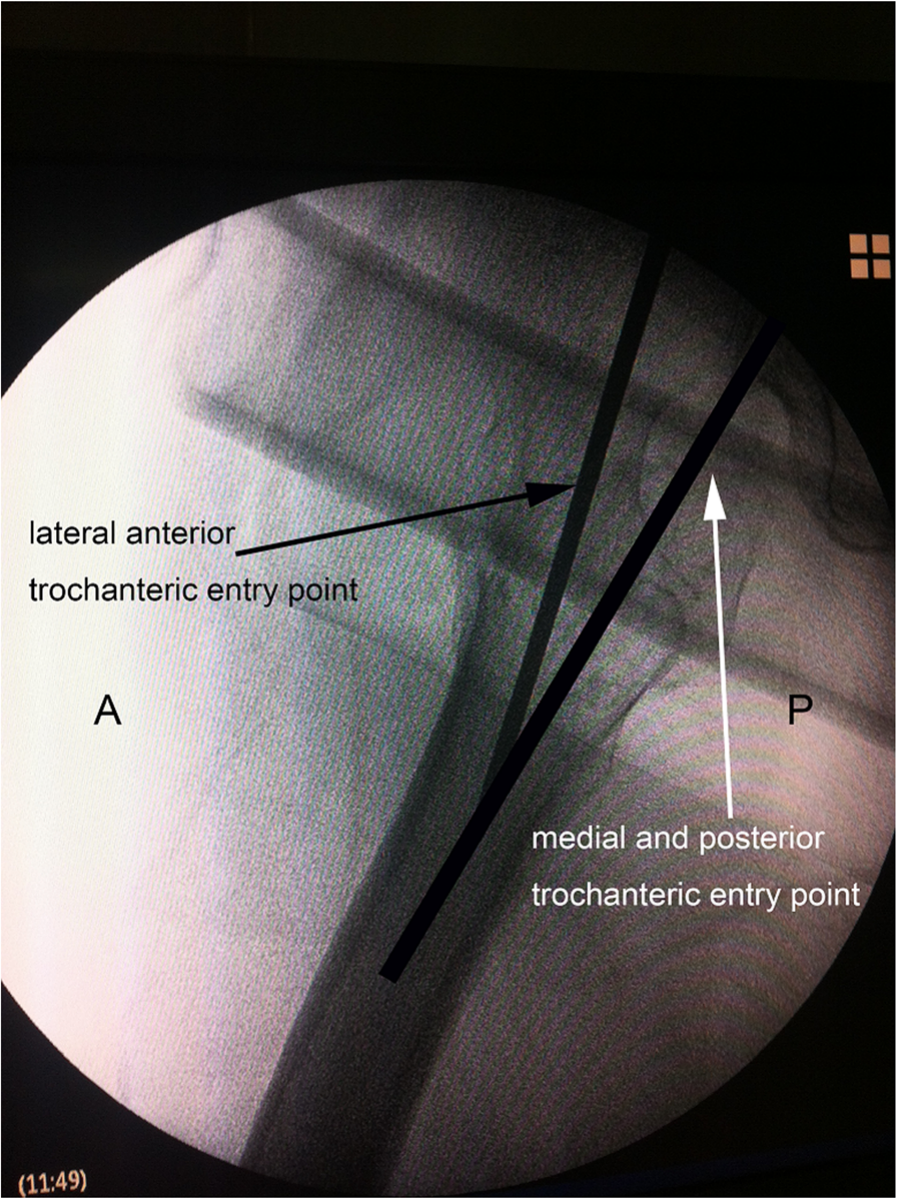
Lateral radiograph. The guided pin insertion entry point in group LA was at the junction of the anterior one-third and posterior two-thirds of the apex of the greater trochanter in the sagittal plane. The guided pin insertion entry point in group MP was 0.5 cm posterior to the apex of the greater trochanter and in the center of the femoral neck

**Fig. 3:**
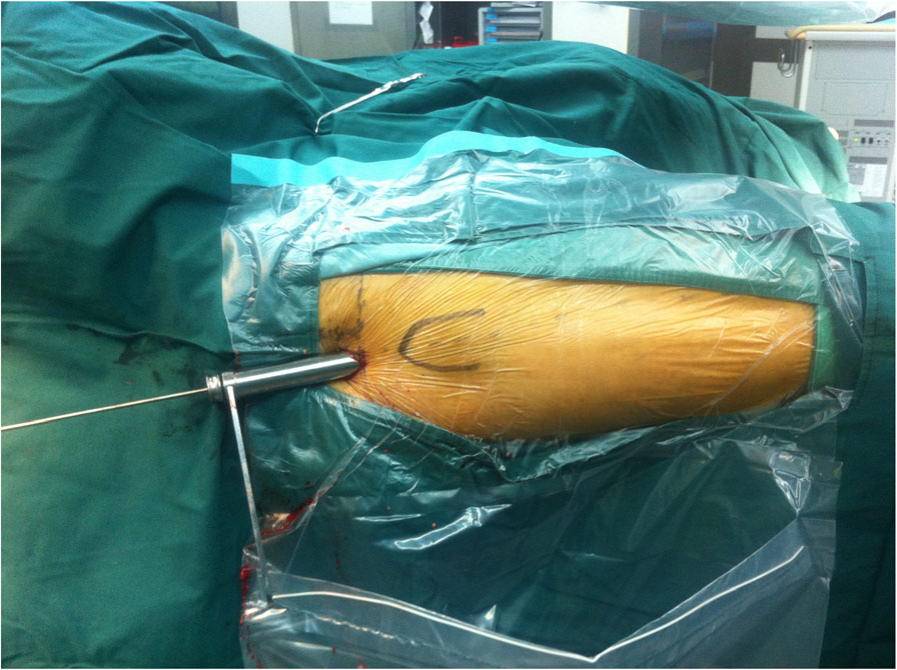
Direction of the guided pin. When the ideal insertion entry point had been identified, the guided pin was inserted along the femoral curvature line at an angle of 30° relative to horizontal

The main difference between the protocols for the two groups is the guide pin entry point of the great trochanter. In group LA (the lateral and anterior trochanteric entry point group), the trochanteric entry point, which was recommended by the manufacturer, was 0.5 cm lateral to the apex of the greater trochanter in the coronal plane and at the junction of the anterior one-third and posterior two-thirds of the apex of the greater trochanter in the sagittal plane. In group MP (the medial and posterior trochanteric entry point group), we currently use a modified trochanteric entry point that is slightly (0.5 cm) posterior and slightly (0.5 cm) medial to the trochanteric apex on the medial edge of the greater trochanter. The guided pin insertion entry point was located 0.5 cm medial to the apex of the greater trochanter on anteroposterior radiographs and 0.5 cm posterior to the apex of the greater trochanter on lateral radiographs (Figs. 1 and 2). When the ideal insertion entry point had been identified, the guided pin was inserted along the femoral curvature line at an angle of 30° relative to the horizontal (Fig. 3). Satisfactory positioning of the guided pin was verified on anteroposterior and lateral radiographs. After reaming of the proximal femur, an appropriate nail was inserted into the femur. Next, the PFNA-II helical blade and distal screw were inserted according to the manufacturer’s recommendations.

### Perioperative management

All patients received antibiotics prophylactically with second-generation cephalosporin 30 min preoperatively and continued for 48 h postoperatively. Patients in both groups were given spinal anesthesia. Subcutaneous low-molecular heparin was continued as thromboembolic prophylaxis for a total of 10 days.

Passive and active hip motion began on postoperative days 1–3 after the drainage had been removed. Partial weight bearing began with the appearance of fracture healing, and total weight bearing began with clinical fracture healing, as assessed with the follow-up radiographs.

All patients underwent follow-up assessments at 1, 3, 6 and 12 months after surgery. Anteroposterior and lateral X-rays, physical examinations, and clinical follow-up data were obtained at each follow-up assessment.

**Fig. 4:**
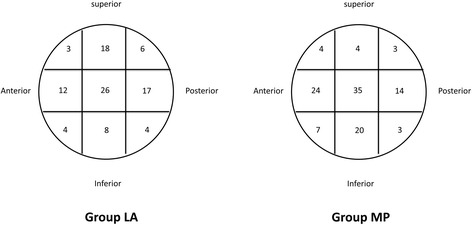
Helical blade position within the femoral head (group LA and group MP)

Radiographic assessments were recorded: The positioning of the nail in the femoral canal was evaluated, and a distance of zero to the lateral trochanteric wall on the anteroposterior view was defined as impingement. The tip-apex distance (TAD) was assessed according to the system of Baumgaertner et al. [15], based on the postoperative anteroposterior and lateral radiographs. As described by Cleveland et al. [16], all of the postoperative radiographs were assessed to determine the position of the helical blade within the femoral head (Fig. 4). Fracture union was defined as the presence of visible bone trabeculae between bone fragments on anteroposterior and lateral radiographs, with the ability to bear full weight on the extremity.

The following clinical data were recorded: surgical time, fluoroscopy usage time, blood loss amounts, visual analogue scale (VAS), thigh pain, length of hospital stay after the surgery, and Harris hip score.

Surgical time, fluoroscopy time, intraoperative and postoperative blood loss were recorded by the nurses who were not involved in the study. Radiographic and clinical functional assessments were performed and recorded by two senior orthopedics that were blinded and not involved in the study.

### Statistical analysis

Descriptive statistics generated using SPSS 20.0 (SPSS, Inc., Chicago, IL, USA) were utilized for data analysis. Kolmogorov–Smirnov tests were used to evaluate the Gaussian distributions of continuous variables. Comparisons of the 2 groups were performed with Student’ T-tests for continuous variables with Gaussian distributions and Mann–Whitney U tests for continuous variables that did not show Gaussian distributions. For categorical variables, Pearson chi-square tests and Fisher’s exact tests were used to evaluate the significance of differences. All *P*-values were 2-sided, and *P*-values below 0.05 were considered significant.

## Results

Two hundred and ninety-six cases of osteoporotic intertrochanteric fractures were treated with PFNA-II in our hospital between May 2010 and October 2015. Eighty-four patients who did not meet the inclusion or meet the exclusion criteria were excluded. Ninety-eight patients treated with the lateral anterior trochanteric entry point (group LA) and 114 patients treated with the medial posterior trochanteric entry point (group MP) included in the study. Table 1 presents the demographic data for each group. The average patient age were 76.64 years for group LA and 76.52 years for group MP (*P* = 0.990). 68 patients (69.3%) in group LA and 71 patients (62.3%) in group MP were female (*P* = 0.278). The ASA and fracture side in two groups were not different statistically (*P* = 0.606, and *P* = 0.853, respectively). The average harris hip score before injury were 91.70 for group LA and 92.33 for group MP (*P* = 0.585). The AO/OTA fracture classification was A1.1 1.2 1.3 2.1 in 63 cases (64.3%), A2.2 2.3 3 in 35 cases (35.7%) for group LA, and A1.1 1.2 1.3 2.1 in 67 cases (58.8%), A2.2 2.3 3 in 35 cases (41.2%) for group MP (*P* = 0.411).

The radiographic reviews are presented in Table 2. The distances to the lateral trochanteric wall were classified as zero – impingement (35 in group LA [33.3%] and 23 in group MP [20.2%]) or as greater than zero – non-impingement (63in group LA [64.3%] and 91 in group MP [79.8%]), and the difference in the classifications between the 2 groups was significant (*P* = 0.011). The TADs in group LA (22.40 ± 4.43) and group MP (23.60 ± 3.60) did not significantly differ (*P* = 0.075). The helical blade positions were recorded and are shown in Fig. 4. There were no significant differences between the 2 groups in the helical blade position in the helical blade position between the 2 groups on the lateral radiographs (*P* = 0.071) However, there was a significant difference in the helical blade position between the 2 groups overall or on the anteroposterior radiographs (*P* = 0.006 and *P* = 0.001, respectively).

**Table 2:** Radiographic review

	Group LA (*n* = 98)	Group MP (*n* = 114)	*P* value
impingement with the lateral trochanteric wall			0.011 ^a^
impingement	35(33.3%)	23(20.2%)	
Non-impingement	63(64.3%)	91(79.8%)	
TAD	22.40 ± 4.43	23.39 ± 3.60	0.075^b^
Helical blade position in all			0.006^c^
Helical blade position(anteroposterior)			0.001^a^
Superior (1.2.3)	28(28.6%)	11(9.6%)	
Middle (4.5.6)	55(56.1%)	73 (64.0%)	
Inferior (7.8.9)	15(15.3%)	30(26.3%)	
Helical blade position(lateral)			0.071^a^
Anterior (1.4.7)	18(18.4%)	35(30.7%)	
Middle (2.5.8)	54(55.1%)	59 (51.8%)	
Posterior (3.6.9)	26(26.5%)	20(17.5%)	

TAD tip-apex distance

^a^Chi-square test

^b^Student’ *T* test

^c^Fisher’s Exact Test

The operative and clinical outcomes are listed in Table 3. The differences in surgical time, hospital stay, and intraoperative and postoperative blood loss between the 2 groups were not significant (*P* > 0.05). The fluoroscopy time of group LA was significantly shorter than that of group MP (56.86 ± 13.24 vs. 63.29 ± 11.12, respectively, *P* = 0.000). Two iatrogenic lateral proximal fractures and 2 helical blade cutouts occurred in group LA, whereas none occurred in group MP. Additionally, the Harris hip scores at 6 and 12 months were not significantly different (*P* = 0.110 and *P* = 0.773, respectively). However, at postoperative months 1 and 3, the Harris hip scores of group MP (60.92 ± 9.71 and 69.35 ± 6.65, respectively) were significantly higher than those of group LA (56.09 ± 12.04 and 63.98 ± 8.06; *P* = 0.001 and *P* = 0.000, respectively). Simultaneously, the differences between the VAS scores at 6 and 12 months were not significant (*P* = 0.653 and *P* = 00.145 respectively); at both 1 and 3 months, group MP (4.80 ± 1.35 and 3.61 ± 1.63, respectively) exhibited lower scores than group LA (5.50 ± 1.25 and 4.21 ± 1.52, respectively, both *P* < 0.05). Nine 240-mm, seventeen 200-mm, and seventy-two 170-mm PFNA-II nails were employed in group LA, and 11, 24, and 79 nails of the same lengths were used in group MP, respectively. The incidence rates of thigh pain in groups that employed the same nail lengths were was similar; however the 240-mm nail groups had higher thigh pain incidence rates (11.11% in group LA and 27.27% in group MP).

**Table 3:** Operative and clinical outcomes

		Group LA (*n* = 98)	Group MP (*n* = 114)	*P* value
surgical time(minutes)		53.26 ± 14.44	49.72 ± 16.79	0.104^a^
fluoroscopy time(seconds)		56.86 ± 13.24	63.29 ± 11.12	0.000^a^
intraoperative blood loss(ml)		109.60 ± 29.59	102.39 ± 24.23	0.053^a^
postoperative blood loss(ml)		77.38 ± 22.32	73.26 ± 25.02	0.211^a^
VAS Score	1 month	5.50 ± 1.25	4.80 ± 1.35	0.000^a^
	3 month	4.21 ± 1.52	3.61 ± 1.63	0.006^a^
	6 month	2.63 ± 1.67	2.54 ± 1.48	0.653^a^
	12 month	2.08 ± 1.19	1.85 ± 1.11	0.145^a^
Thigh pain	240 mm	1(9)11.11%	3(11)27.27%	0.591^b^
	200 mm	1(17)5.88%	2(24)8.33%	1.000^b^
	170 mm	2(72)2.78%	5(79)6.33%	0.446^b^
Harris Hip Score	1 month	56.09 ± 12.04	60.92 ± 9.71	0.001^a^
	3 month	63.98 ± 8.06	69.35 ± 6.65	0.000^a^
	6 month	73.38 ± 8.59	75.22 ± 8.08	0.110^a^
	12 month	78.53 ± 8.77	78.16 ± 9.83	0.773^a^
hospital stay(day)		10.71 ± 4.00	11.53 ± 4.29	0.156^a^

^a^Student’ *T* test

^b^Fisher’s Exact Test

## Discussion

There are many studies related to the treatment of intertrochanteric fracture with PFNA, most of which focus on instrument characteristics [2, 4, 5, 17–21]. With further research and development, more investigators have found that the surgical technique (particularly the entry point) plays an important role in the clinical outcome [8, 10–12, 22, 23]. To our knowledge, this study is the first to compare the radiographic and clinical characteristics between two types of trochanteric entry points for PFNA. In this investigation, we found that in the postoperative radiographs of group MP, fewer impingements on the lateral trochanteric wall were noted, the nail positions were deeper, and the helical blade positions in the femoral head were lower. At 1 and 3 months, group MP had a better clinical outcome, with lower VAS scores and higher Harris hip scores than group LA. However, the fluoroscopy time of group LA was shorter than that of group MP.

The lateral trochanteric wall, which buttresses the proximal fragment, has been recognized as an important predictor of stability in intertrochanteric fractures, and fracture of the lateral trochanteric wall yields a high risk of implant failure [24, 25]. Despite the fact that an IM nail provides high union rates with low major complication rates, it has been associated with lateral trochanteric wall impingement that causes lateral trochanteric wall fracture during insertion, particularly for Asian patients with a narrow and short proximal femoral anatomy [17, 19, 21, 26–28]. PFNA II has been improved for the Asian patient with a flattened lateral surface, and the decreased mediolateral nail angle tends to reduce the risk of impingement with the lateral trochanteric wall [6, 19, 29]. However, a geometric mismatch of PFNA II to the Asian proximal femoral anatomy has also been reported. Tyagi et al. [18], in a study based on computer tomography, reported that the Asian proximal-femoral average bending angle in the coronal plane is 8.4 ± 2.2°, and 80% are greater than the 5° bending angle of PFNA II. Consequently, impingement with the lateral trochanteric wall is not a rare phenomenon when a trochanteric tip entry point is employed. In our study, we observed 35 of 98 patients (33.3%) with impingement in group LA, in which the entry point in the coronal plane was located at the intertrochanteric tip; in the MP group (with a medial entry point), impingement was observed in 23 of 114 patients (20.2%), and this difference was significant. Impingement results in high stress on the lateral trochanteric wall and causes an iatrogenic fracture in which the cortical bone is weakened. In group LA, there were 5 cases of iatrogenic fractures with poor early function. Similarly, Zhang et al. [30] also reported 1 case of iatrogenic fracture caused by insertion of the PFNA II nail (which employed the tip of intertrochanteric as the entry point) in 56 patients.

Currently, the main complication of PFNA is helical blade cutout and penetration [2, 7, 31, 32]. Although a helical blade achieves an excellent fit through bone compaction around the blade to avoid rotation and varus collapse, an undesirable helical blade position resulting in implant complication/failure occurs more often in elderly Asians with osteoporosis [31, 33–36]. The TAD is widely recognized as an essential index for predicting fixation failure with lag screws or blade cutouts from the femoral head following surgical fixation with IM nailing [16]. The latest biomechanical research suggests that the TAD for the PFNA should be 20–30 mm and that the blade position should be low in the anteroposterior plane and in the center of the lateral plane to achieve the best stability [37, 38]. Because the femoral geometry of Asians involves shorter femoral necks, the smaller femoral neck angles and shorter proximal canals increase the difficultly of obtaining the ideal blade position, particularly in elderly Asian women [39–41]. In this study, only 75% reached the ideal position; three cases of cutout occurred in group LA due to an excessively high helical blade position. To insert the blade lower in the anteroposterior plane, the nail must be inserted deeply, which causes the nail to impinge on the lateral cortex in patients with short proximal canals. Simultaneously, small femoral neck angles also result in higher blade positions with a high risk of cutout. In the present study, the TADs of group MP were slightly greater than those of group LA, but this difference was not significant. In the Cleveland zone, the blade position was lower in group MP than in group LA, and 3 cutouts requiring revision surgery occurred in group LA. We believe that the use of an entry point slightly medial to the trochanteric tip allowed the nail to be inserted deeper and more medially, which in turn enabled the helical blade to be placed at a lower position in the femoral head.

The entry point of the PFNA in the sagittal plane remains controversial. Some researchers suggest that the entry point should be at the junction of the anterior one-third and posterior two-thirds of the apex of the greater trochanter. However, recent cadaver research has indicated that the apex of the greater trochanter is consistently anterior to the IM canal [11]. Therefore, to accommodate the IM canal, the entry point of a short, straight PFNA should be approximately 0.5 cm posterior to the apex of the greater trochanter, and the nail should be inserted at a 30° angle with respect to the horizontal [11, 42]. However, with a posterior entry point, some studies have indicated that setting the helical blade properly within the femoral head is difficult and that the blade will be cut out. Furthermore, the nail requires additional external rotation to allow the helical blade to be set into the femoral head. Similarly, in this study, we found that group MP (with the posterior entry point) needed more fluoroscopy time to avoid guide pin in-out-in within the femoral head. However, regarding the helical blade position in the femoral head, we observed no significant differences between the 2 groups in the lateral radiographs (*P* = 0.071), and no cutouts occurred in group MP. We believe the above finding was due to moving the entry point medially, which allowed the nail to be inserted deeper and the helical blade to assume a lower position.

Chang et al. [29] reported that a mismatch between PFNA II and the medullary canal causes an impingement of the anterior femoral cortex, which yields thigh pain and even femoral shaft fractures. Our research also revealed that thigh pain occurred in 20% of patients with a 240-mm nail and in only 7.32% and 4.64% of patients with 200-mm and 170-mm nails, respectively, but there were no femoral fractures. The thigh pain in the two groups with different entry points was not significant. Theoretically, an anterior entry point will cause less femoral cortex impingement, but in this study, the difference was not significant. The reason is that, with a posterior entry point, the nail requires external rotation to achieve the correct helical blade position in the femoral head, which turns the lateral bending of the nail into the sagittal plane, and then the tip of the nail assumes a posterior position.

In the present study, we observed that the Harris hip score in group MP was significantly greater than in group LA at 1 and 3 months postoperatively, while at 6 and 12 months after surgery, there was no significant difference between the groups with respect to the Harris hip score. McConnell et al. [14] emphasized that the lateral trochanteric point caused an average of 27% damage to the gluteus medius tendon during the reaming for IM nail insertion. These authors proposed that gluteus medius tendon injury should be recognized as a cause of postoperative morbidity. In a cadaver study, Perez et al. [43] described the entry point as being slightly medial to the trochanteric apex along the trochanteric ridge and did not damage the gluteus medius tendon during reaming. Tao et al. [39] also suggested that a medial entry point can decrease postoperative morbidity. Therefore, the difference in the Harris hip score between the two groups may be due to the damage to the gluteus medius tendon in group LA; after 6 months, the gluteus medius tendon was fully recovered, which made the score increase. An additional study should be conducted to prove this point.

While group MP received good clinical outcomes, the fluoroscopy time was longer than that of group LA. Achieving a medial entry point requires more fluoroscopy because the guide pin always slides into the pyriform fossa from the trochanteric ridge. Furthermore, group MP (with a posterior entry point) required more fluoroscopy to confirm the helical blade position in the femoral head. Achieving the LA entry point required less fluoroscopy time and an easier operation; however, this difference in fluoroscopy time did not cause a significant difference in the operation time.

This study has several limitations. First, obtaining precise positioning of the entry point during the surgery was difficult, particularly with greater trochanteric fractures, even after repeated fluoroscopy; as a future direction of research, we are considering the use of intraoperative CT or navigation systems to pinpoint the entry. Second, all of the patients in this study were local residents of northern China; thus, whether our results can be generalized to southern Chinese individuals with shorter statures requires further multicenter studies. Third, in this study, perfect anteroposterior and lateral views were difficult to achieve during the intraoperative fluoroscopy. However, Farhang et al.’s [11] research (a recent anatomical study) revealed that mild proximal-femoral rotation does not significantly affect the placement of the trochanteric entry point. Therefore, we suggest that future studies with long-term follow-ups, multicenter evaluations, and accurate analyses with computer tomography are necessary.

## Conclusions

This retrospective cohort study demonstrated that use of the medial posterior trochanteric entry point results in early hip function recovery and achieves excellent nail positions with fewer impingements, a lower helical blade position, and fewer surgical complications. However, the medial posterior trochanteric entry point was more difficult to utilize than the lateral anterior trochanteric entry point, and longer fluoroscopy time was required.

## Abbreviations

ASA: American Society of Anesthesiologists
IM: Intramedullary
PFNA: Proximal femoral nail antirotation
SPMSQ: Short Portable Mental Status Questionnaire
TAD: Tip-apex distance
VAS: Visual analogue scale

## References

[1] Endo Y, Aharonoff GB, Zuckerman JD, Egol KA, Koval KJ (2005). Gender differences in patients with hip fracture: a greater risk of morbidity and mortality in men. J Orthop Trauma.

[2] Simmermacher RK, Ljungqvist J, Bail H, Hockertz T, Vochteloo AJ, Ochs U (2008). The new proximal femoral nail antirotation (PFNA) in daily practice: results of a multicentre clinical study. Injury.

[3] Wild M, Jungbluth P, Thelen S, Laffrée Q, Gehrmann S, Betsch M, et al. The dynamics of proximal femoral nails: a clinical comparison between PFNA and Targon PF. Orthopedics. 2010;33: doi:10.3928/01477447-20100625-04. 10.3928/01477447-20100625-04 20704115

[4] Kristek D, Lovrić I, Kristek J, Biljan M, Kristek G, Sakić K (2010). The proximal femoral nail antirotation (PFNA) in the treatment of proximal femoral fractures. Coll Antropol.

[5] Seyhan M, Turkmen I, Unay K, Ozkut AT (2015). Do PFNA devices and Intertan nails both have the same effects in the treatment of trochanteric fractures? A prospective clinical study. J Orthop Sci.

[6] Sawaguchi T, Sakagoshi D, Shima Y, Ito T, Goldhahn S (2014). Do design adaptations of a trochanteric nail make sense for Asian patients? Results of a multicenter study of the PFNA-II in Japan. Injury.

[7] Liu Y, Tao R, Liu F, Wang Y, Zhou Z, Cao Y (2010). Mid-term outcomes after intramedullary fixation of peritrochanteric femoral fractures using the new proximal femoral nail antirotation (PFNA). Injury.

[8] Anastopoulos G, Chissas D, Dourountakis J, Ntagiopoulos PG, Magnisalis E, Asimakopoulos A (2010). Computer-assisted three-dimensional correlation between the femoral neck-shaft angle and the optimal entry point for antegrade nailing. Injury.

[9] Ansari Moein CM, Ten Duis HJ, Oey PL, de Kort GA, van der Meulen W, van der Werken C (2011). Intramedullary femoral nailing through the trochanteric fossa versus greater trochanter tip: a randomized controlled study with in-depth functional outcome results. Eur J Trauma Emerg Surg.

[10] Crookshank MC, Edwards MR, Sellan M, Whyne CM, Schemitsch EH (2014). Can fluoroscopy-based computer navigation improve entry point selection for intramedullary nailing of femur fractures?. Clin Orthop Relat Res.

[11] Farhang K, Desai R, Wilber JH, Cooperman DR, Liu RW (2014). An anatomical study of the entry point in the greater trochanter for intramedullary nailing. Bone Joint J.

[12] Zhao JX, Su XY, Zhao Z, Zhang LC, Mao Z, Zhang H (2015). Predicting the optimal entry point for femoral antegrade nailing using a new measurement approach. Int J Comput Assist Radiol Surg.

[13] Ziran BH, Morganstein A (2014). Preventing eccentric reaming of the trochanter during trochanteric nailing. J Orthop Trauma.

[14] McConnell T, Tornetta P, Benson E, Manuel J (2003). Gluteus medius tendon injury during reaming for gamma nail insertion. Clin Orthop Relat Res.

[15] Baumgaertner MR, Curtin SL, Lindskog DM, Keggi JM (1995). The value of the tip-apex distance in predicting failure of fixation of peritrochanteric fractures of the hip. J Bone Joint Surg Am.

[16] Cleveland M, Bosworth DM, Thompson FR (1947). Intertrochanteric fractures of the femur; a survey of treatment in traction and by internal fixation. J Bone Joint Surg Am.

[17] Pu JS, Liu L, Wang GL, Fang Y, Yang TF (2009). Results of the proximal femoral nail anti-rotation (PFNA) in elderly Chinese patients. Int Orthop.

[18] Tyagi V, Yang JH, Oh KJ (2010). A computed tomography-based analysis of proximal femoral geometry for lateral impingement with two types of proximal femoral nail anterotation in subtrochanteric fractures. Injury.

[19] Macheras GA, Koutsostathis SD, Galanakos S, Kateros K, Papadakis SA (2012). Does PFNA II avoid lateral cortex impingement for unstable peritrochanteric fractures?. Clin Orthop Relat Res.

[20] Zhang S, Zhang K, Wang Y, Feng W, Wang B, Yu B (2013). Using three-dimensional computational modeling to compare the geometrical fitness of two kinds of proximal femoral intramedullary nail for Chinese femur. ScientificWorldJournal.

[21] Sahin EK, Imerci A, Kınık H, Karapınar L, Canbek U, Savran A (2014). Comparison of proximal femoral nail antirotation (PFNA) with AO dynamic condylar screws (DCS) for the treatment for unstable peritrochanteric femoral fractures. Eur J Orthop Surg Traumatol.

[22] Gausepohl T, Pennig D, Koebke J, Harnoss S (2002). Antegrade femoral nailing: an anatomical determination of the correct entry point. Injury.

[23] Amarathunga JP, Schuetz MA, Yarlagadda KVD, Schmutz B (2015). Is there a bone-nail specific entry point? Automated fit quantification of tibial nail designs during the insertion for six different nail entry points. Med Eng Phys.

[24] Marsh JL, Slongo TF, Agel J, Broderick JS, Creevey W, DeCoster TA (2007). Fracture and dislocation classification compendium - 2007: Orthopaedic Trauma Association classification, database and outcomes committee. J Orthop Trauma.

[25] Kim Y, Bahk WJ, Yoon YC, Cho JW, Shon WY, Oh CW (2015). Radiologic healing of lateral femoral wall fragments after intramedullary nail fixation for A3. 3 intertrochanteric fractures. Arch Orthop Trauma Surg.

[26] Sahin S, Ertürer E, Oztürk I, Toker S, Seçkin F, Akman S (2010). Radiographic and functional results of osteosynthesis using the proximal femoral nail antirotation (PFNA) in the treatment of unstable intertrochanteric femoral fractures. Acta Orthop Traumatol Turc.

[27] Han N, Sun GX, Li ZC, Li GF, Lu QY, Han QH (2011). Comparison of proximal femoral nail antirotation blade and reverse less invasive stabilization system-distal femur systems in the treatment of proximal femoral fractures. Orthop Surg.

[28] Guo Q, Shen Y, Zong Z, Zhao Y, Liu H, Hua X (2013). Percutaneous compression plate versus proximal femoral nail anti-rotation in treating elderly patients with intertrochanteric fractures: a prospective randomized study. J Orthop Sci.

[29] Chang SM, Song DL, Ma Z, Tao YL, Chen WL, Zhang LZ (2014). Mismatch of the short straight cephalomedullary nail (PFNA-II) with the anterior bow of the femur in an Asian population. J Orthop Trauma.

[30] Zhang S, Zhang K, Jia Y, Yu B, Feng W (2013). InterTan nail versus proximal femoral Nail Antirotation-Asia in the treatment of unstable trochanteric fractures. Orthopedics.

[31] Cheung JP, Chan CF (2011). Cutout of proximal femoral nail antirotation resulting from blocking of the gliding mechanism during fracture collapse. J Orthop Trauma.

[32] Soucanye de Landevoisin E, Bertani A, Candoni P, Charpail C, Demortiere E (2012). Proximal femoral nail antirotation (PFN-ATM) fixation of extra-capsular proximal femoral fractures in the elderly: retrospective study in 102 patients. Orthop Traumatol Surg Res.

[33] Niikura T, Lee SY, Matsumoto T, Fukui T, Kawakami Y, Akisue T (2012). Backout of the helical blade of proximal femoral nail antirotation and accompanying fracture nonunion. Orthopedics.

[34] Lee SY, Niikura T, Iwakura T, Sakai Y, Kuroda R, Kurosaka M (2014). Complete traumatic backout of the blade of proximal femoral nail antirotation: a case report. Orthop Traumatol Surg Res.

[35] Takigami I, Ohnishi K, Ito Y, Nagano A, Sumida H, Tanaka K (2011). Acetabular perforation after medial migration of the helical blade through the femoral head after treatment of an unstable trochanteric fracture with proximal femoral nail antirotation (PFNA): a case report. J Orthop Trauma.

[36] Brunner A, Büttler M, Lehmann U, Frei HC, Kratter R, Di Lazzaro M (2016). What is the optimal salvage procedure for cut-out after surgical fixation of trochanteric fractures with the PFNA or TFN?: a multicentre study. Injury.

[37] Kane P, Vopat B, Heard W, Thakur N, Paller D, Koruprolu S (2014). Is tip apex distance as important as we think? A biomechanical study examining optimal lag screw placement. Clin Orthop Relat Res.

[38] Nikoloski AN, Osbrough AL, Yates PJ (2013). Should the tip-apex distance (TAD) rule be modified for the proximal femoral nail antirotation (PFNA)? A retrospective study. J Orthop Surg Res.

[39] Tao Y, Ma Z, Chang S (2013). Does PFNA II avoid lateral cortex impingement for unstable peritrochanteric fractures?. Clin Orthop Relat Res.

[40] Chin K, Evans MC, Cornish J, Cundy T, Reid IR (1997). Differences in hip axis and femoral neck length in premenopausal women of Polynesian, Asian and European origin. Osteoporos Int.

[41] Cummings SR, Cauley JA, Palermo L, Ross PD, Wasnich RD, Black D (1994). Racial differences in hip axis lengths might explain racial differences in rates of hip fracture. Study of Osteoporotic Fractures Research Group. Osteoporos Int.

[42] Hwang JH, Oh JK, Han SH, Shon WY, Oh CW (2008). Mismatch between PFNa and medullary canal causing difficulty in nailing of the pertrochanteric fractures. Arch Orthop Trauma Surg.

[43] Perez EA, Jahangir AA, Mashru RP, Russell TA (2007). Is there a gluteus medius tendon injury during reaming through a modified medial trochanteric portal? A cadaver study. J Orthop Trauma.

